# Evaluation of transverse maxillary growth on cone-beam computed tomography images

**DOI:** 10.1038/s41598-021-97082-0

**Published:** 2021-08-31

**Authors:** Brandon J. Seubert, Laurence Gaalaas, Brent E. Larson, Thorsten Grünheid

**Affiliations:** 1 Private Practice, Rochester, MN USA; 2 Division of Oral Medicine, Diagnosis and Radiology, School of Dentistry, University of Minnesota, Minneapolis, MN USA; 3 Division of Orthodontics, School of Dentistry, University of Minnesota, 6-320 Moos Health Science Tower, 515 Delaware Street S.E., Minneapolis, MN 55455 USA

**Keywords:** Orthodontics, Bone

## Abstract

This study aimed at quantifying the annual transverse growth of the maxilla using skeletal landmarks in three different regions on cone-beam computed tomography (CBCT) scans. CBCT scans taken before and after orthodontic treatment of 100 child and adolescent patients (50 male, 50 female) without maxillary transverse deficiencies were used to determine the transverse linear distances between the greater palatine foramina (GPFd), the lateral walls of the nasal cavity (NCd), and the infraorbital foramina (IOFd). We found that all distances increased significantly with growth in both genders (*p* < 0.001). The overall average annual change was 0.5 mm for GPFd, 0.3 mm for NCd, and 0.7 mm for IOFd. Males generally had greater annual changes than females for GPFd and IOFd, but not NCd. There were weak, statistically not significant (*p* > 0.05) correlations between patient age and the annual changes in GPFd, NCd, and IOFd. These results suggest that the positions of the greater palatine foramina, the lateral walls of the nasal cavity, and the infraorbital foramina change consistently with maxillary transverse growth. Clinicians can use the growth rates as population averages to more confidently estimate the amount of skeletal transverse deficiency or evaluate the long-term effects of maxillary expansion treatment.

## Introduction

For many decades, orthodontists have used two-dimensional radiographic imaging and cephalometry to assess the relationships of the maxillofacial skeleton and the dentition in their patients, including evaluation of growth and development, diagnosis, treatment planning, and assessment of treatment progress and outcomes. However, the limitations of lateral radiography have long been realized as many orthodontic problems involve the transverse dimension^1^. Although panoramic, submentovertex, or postero-anterior cephalometric radiographs remain valuable tools to assess the transverse dimension, these imaging modalities suffer from shortcomings such as distortion of the image, magnification errors, and difficulty distinguishing structures due to overlap.

With cone-beam computed tomography (CBCT), it is now possible to visualize the whole maxillofacial complex without any magnification or superimposition of anatomic structures commonly associated with two-dimensional images^2,3^. This allows for accurate and reliable measurement of objects in all planes of space^4–6^. Where regulation permits, orthodontists are increasingly using CBCT scans as the preferred imaging method for diagnosis and treatment planning. Since dedicated CBCT scanners for the oral and maxillofacial region became available almost two decades ago^2^, early adopters of this imaging modality have now accumulated databases of serial craniofacial CBCT scans in healthy individuals. These scans provide an opportunity for characterization of transverse maxillary changes in a longitudinal fashion.

Since growth affect all parts of the maxilla, it is necessary to use landmarks in the inferior, middle, and superior parts of the maxilla to quantify its transverse dimension at various levels. Ideal landmarks would be bilateral and symmetrical, allowing for easy assessment of transverse changes. The obvious choices for such landmarks are maxillary foramina and the nasal cavity. The purpose of this study was to quantify the annual transverse growth of the maxilla using three sets of easily identifiable skeletal landmarks at various vertical levels on CBCT scans to provide normative data on maxillary transverse growth. The landmarks include the lateral margins of the greater palatine foramina, the lateral walls of the nasal cavity, and the lateral margins of the infraorbital foramina. Hitherto, these landmarks have not been used to quantify transverse maxillary growth on 3D images. For each landmark, we tested the null hypothesis that there is no significant change in the transverse dimensions with growth.

## Methods

This retrospective cohort study was approved at the exempt level by the Institutional Review Board at the University of Minnesota (STUDY00008321). All study procedures were performed in accordance with the relevant guidelines and regulations of the University of Minnesota’s Human Research Protection Program. All subjects included in the study or their legal guardians had provided written informed consent for the use of their orthodontic records for research purposes.

The pre-treatment (T1) and post-treatment (T2) CBCT scans of 50 male and 50 female child and adolescent patients who had undergone comprehensive orthodontic treatment with preadjusted edgewise appliances (“braces”) at the University of Minnesota were used. The T1 scans had been taken for diagnosis and treatment-planning purposes while the T2 scans had been taken for outcome assessment purposes. The inclusion criteria were a Class I malocclusion without posterior crossbite or other transverse problems. The exclusion criteria were a history of periodontal disease, congenital malformations, or treatment involving the use of auxiliary appliances such as expanders, headgears, or transpalatal arches. Patient age ranged from 8 to 13 years. Descriptive patient information is summarized in Table 1. All CBCT scans were full field-of-view (17 × 23 cm) scans taken on a dedicated CBCT scanner (i-CAT Next Generation; Imaging Sciences International, Hatfield, PA, USA) at 120 kV and 18.54 mAs, with a pulsed scan time of 8.9 s, and a voxel size of 0.3 mm resulting in a slice thickness of 0.3 mm for all scans. A random sequence generator (random.org) was used to assign the CBCT scans numerical identifiers from 1 to 200 to randomize the order in which measurements were completed and to blind the examiner to the pre- or post-treatment status of the scans.

**Table 1:** Descriptive statistics of patient variables.

Variable	Male (n = 50)		Female (n = 50)		Overall (n = 100)	
	Mean ± SD	Range	Mean ± SD	Range	Mean ± SD	Range
Age at T1 (years)	12.3 ± 0.9	8.4–13.6	11.5 ± 1.1	8.2–12.8	11.9 ± 1.1	8.2–13.6
Age at T2 (years)	14.8 ± 1.0	11.0–16.4	13.9 ± 1.1	10.2–15.7	14.3 ± 1.1	10.2–16.4
T2-T1 (years)	2.5 ± 0.7	1.4–4.3	2.4 ± 0.8	1.3–6.1	2.5 ± 0.8	1.3–6.1
ANB at T1 (°)	2.9 ± 1.4	−0.3–5.5	3.2 ± 1.6	0.1–6.6	3.0 ± 1.5	−0.3–6.6
FMA at T1 (°)	24.2 ± 5.1	15.1–37.2	24.8 ± 5.1	9.5–35.9	24.5 ± 5.1	9.5–37.2

SD: Standard deviation.

ANB: ANB angle.

FMA: Frankfort-mandibular plane angle.

**Figure 1:**
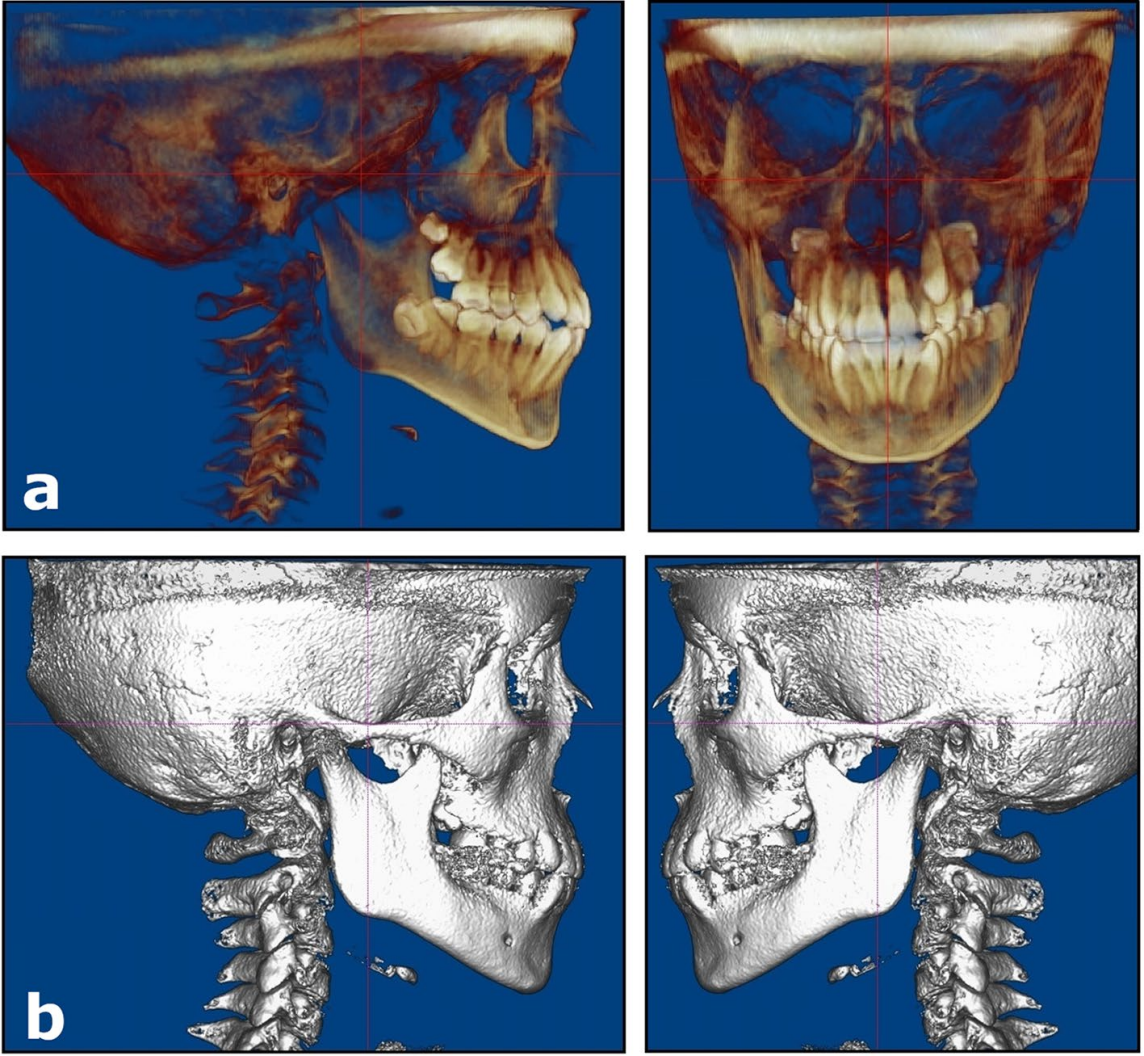
Orientation of the 3D reconstructions in the Dolphin Imaging software. (**a**) Translucent lateral views used to superimpose the orbital rims and the zygomatic processes of the maxilla. (**b**) Solid lateral views used to orient Frankfort Horizontal parallel to the floor.

Transverse linear measurements were made between the three bilateral skeletal landmarks: (1) the greater palatine foramina, (2) the lateral walls of the nasal cavity, and (3) the infraorbital foramina. These landmarks are apical and/or lingual to the dentition and are assumed to be unaffected by the forces generated by the orthodontic appliances used. Measurements were made on digital imaging and communications in medicine (DICOM) images using dedicated imaging software (Dolphin Imaging version 11.9; Dolphin Imaging & Management Solutions, Chatsworth, CA, USA; www.dolphinimaging.com). To ensure that T1 and T2 images were positioned in the same spatial orientation, each CBCT volume was first oriented using translucent lateral views to achieve superimposition of the inferior orbital rims and the zygomatic processes of the maxilla to control yaw and roll (Fig. 1). After that, solid right and left lateral views were used to orient the volume to Frankfort Horizontal, i.e.; a plane joining the upper rims of the external auditory canals and the inferior borders of the orbital rims, to control the pitch.

**Figure 2:**
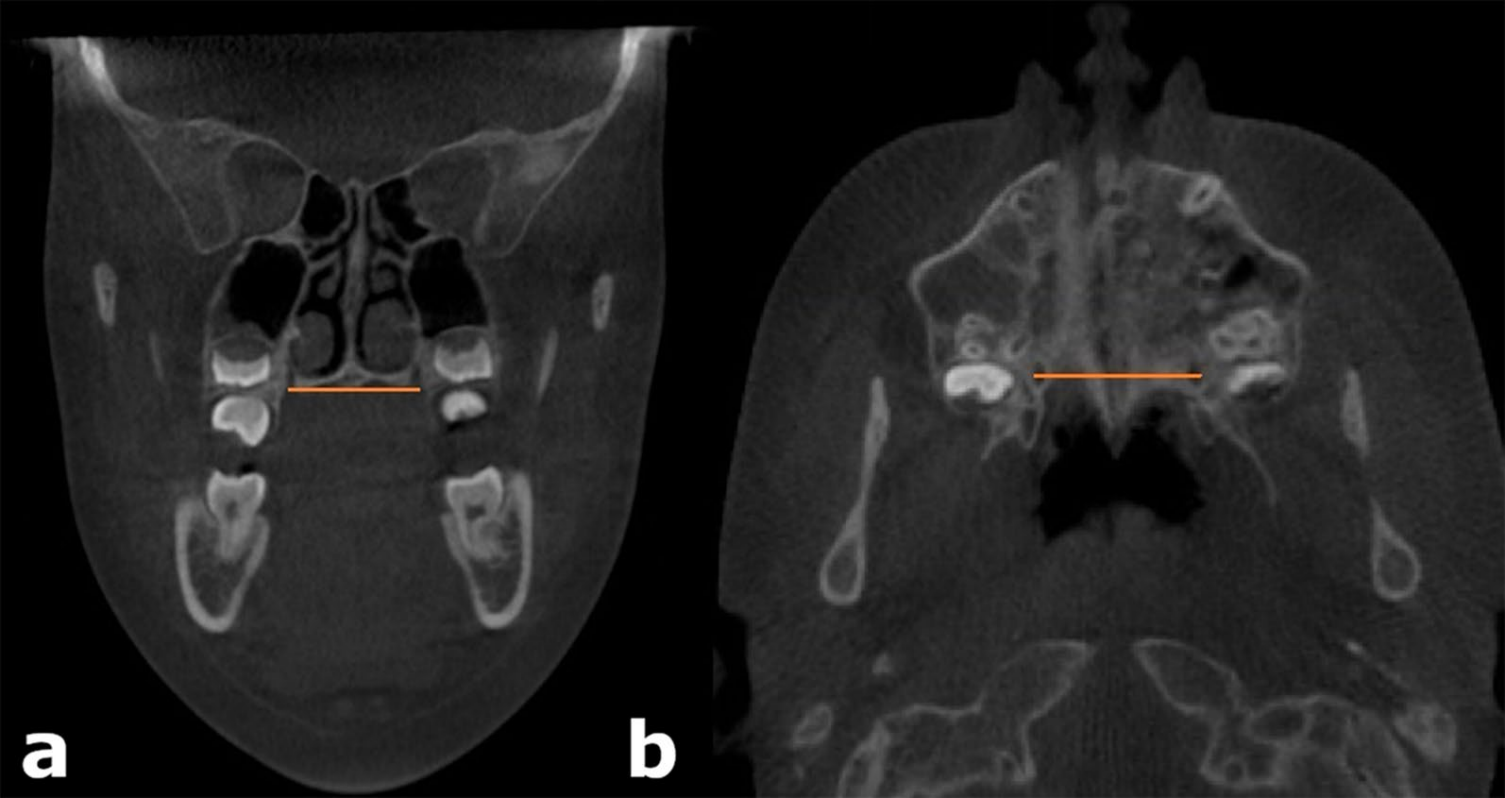
Measurement of the linear distance between the lateral margins of the greater palatine foramina (GPFd). (**a**) Coronal view, (**b**) Axial view.

**Figure 3:**
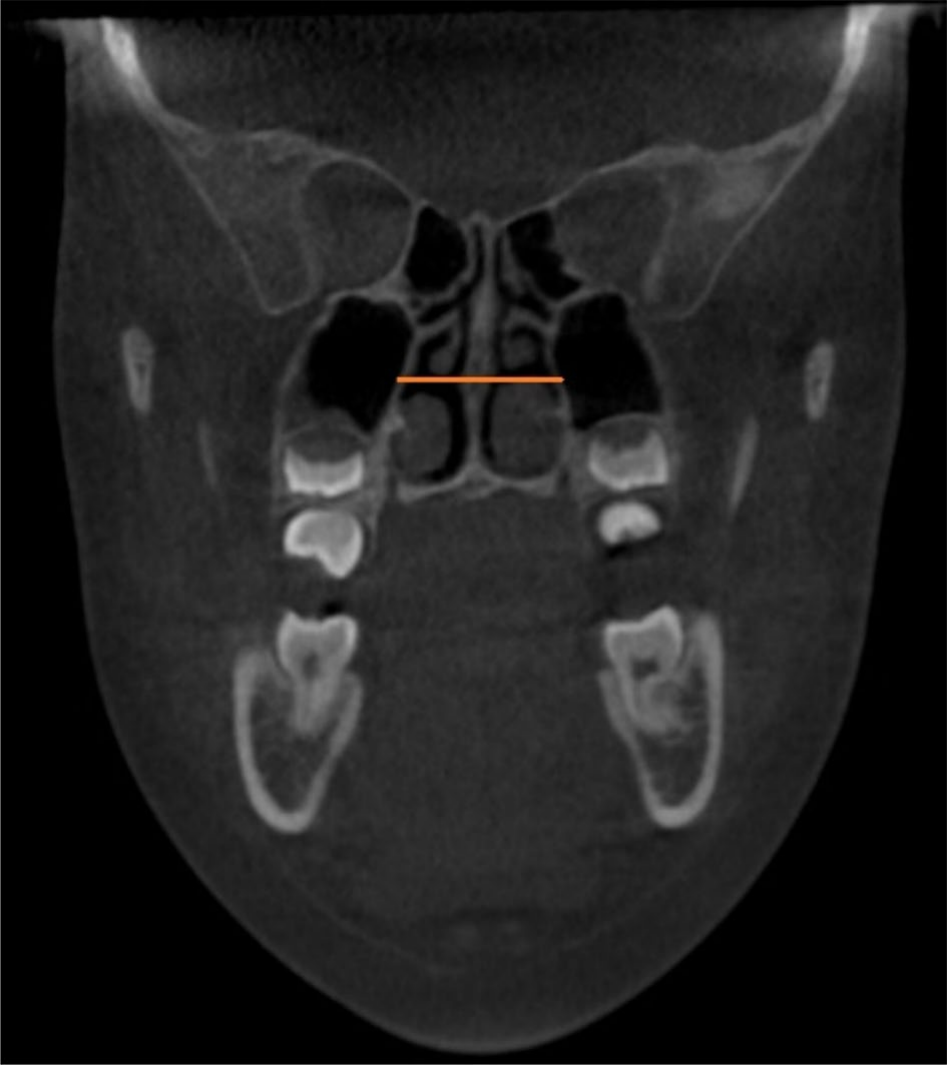
Measurement of the linear distance between the lateral walls of the nasal cavity (NCd), coronal view.

**Figure 4:**
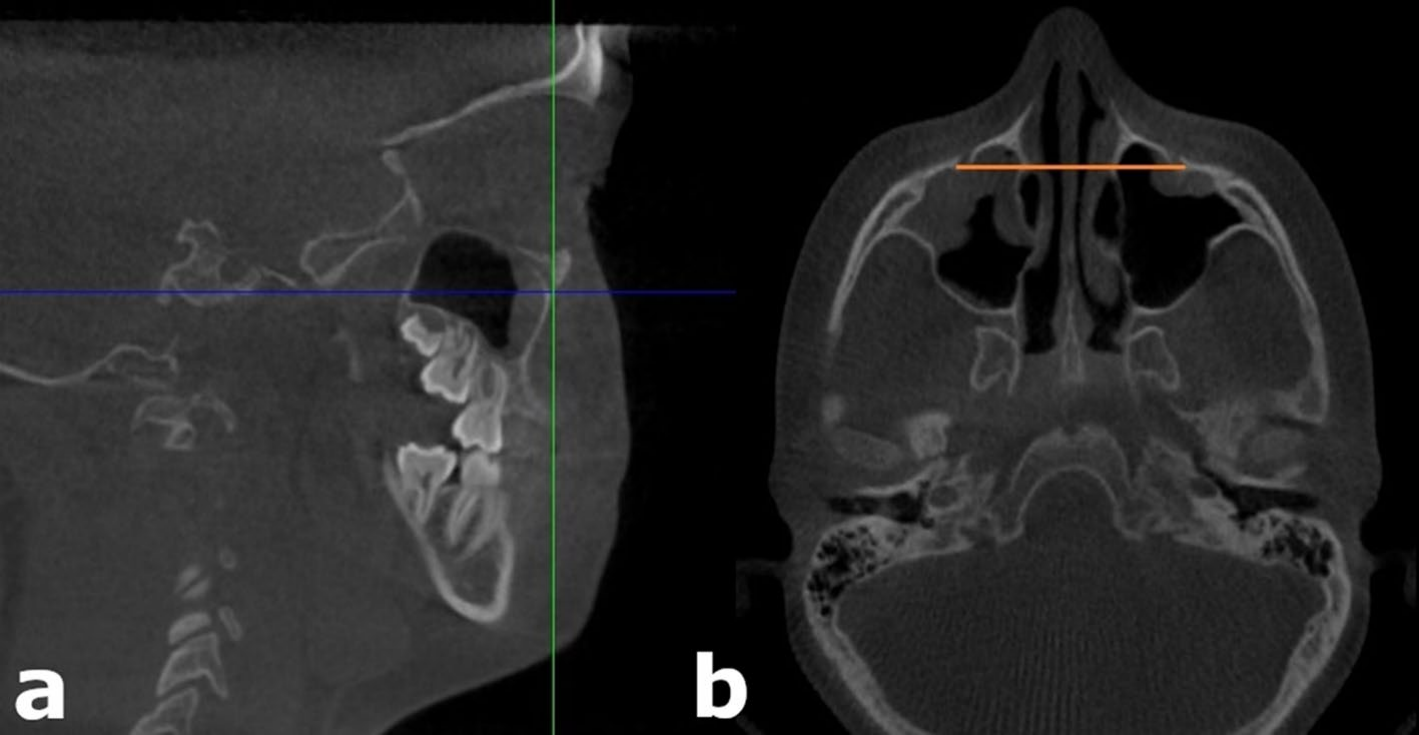
Measurement of the linear distance between the lateral margins of the infraorbital foramina (IOFd). (**a**) Sagittal view, (**b**) Axial view.

The distance between the greater palatine foramina (GPFd) was measured between the lateral margins of the foramina on a coronal slice at the level of the inferior hard palate (Fig. 2). The measurement was verified by viewing it on a corresponding axial slice through the hard palate. The nasal cavity width (NCd) was measured as the distance between the lateral bony walls of the nasal cavity between the middle nasal meatuses at the mucosal crest oriented towards the middle nasal meatus (Fig. 3). The measurement was taken on a coronal slice through the center of the greater palatine foramina—the same slice on which the GPFd was measured. This practice allowed standardization of measurements, which is especially important in patients with asymmetric internal anatomy of the nasal cavity. The distance between the infraorbital foramina (IOFd) was measured between the lateral margins of the foramina on an axial slice after their positions had been identified on a sagittal slice (Fig. 4). All measurements were made to the nearest 0.1 mm by a single examiner (B.S.) and were repeated after a 3-week washout period for 20 randomly chosen subjects to assess intra-examiner reliability.

### Statistical analysis

A sample size calculation based on the results of a subset of 20 subjects evaluated prior to study initiation indicated that a minimum sample size of 30 subjects would be needed to demonstrate statistical significance for each continuous variable with 80% power and a 5% significance level. Descriptive statistics were calculated for all variables. Intra-examiner reliability was assessed using intraclass correlation coefficients. To evaluate changes associated with growth, the distances measured on T1 CBCT images were subtracted from the distances measured on the corresponding T2 CBCT images. Subsequently, the annual change was calculated for all variables (GPFd, NCd, and IOFd) as the difference between the respective T1 and T2 measurements in millimeters divided by the time interval between T1 and T2 CBCT scan in weeks multiplied by 52. One-sample t-tests were used to evaluate whether the average annual changes were statistically significantly different from zero. Two-sample t-tests were used to compare the annual changes between males and females. Parametric tests were chosen after the normality of the data had been confirmed. Pearson’s correlation coefficients were calculated to examine the relationships between the continuous variables. Statistical analyses were performed using SAS 9.4 (SAS 9.4 for Windows version 9.4; SAS Institute, Cary, NC, USA) with *p*-values of less than 0.05 considered statistically significant.

## Results

Intraclass correlation coefficients for GPFd, NCd, and IOFd were 0.994, 0.984, and 0.992, respectively, indicating excellent intra-examiner reliability^7^.

Mean values, standard deviations, and ranges of the dependent variables at T1 and T2 are shown in Table 2. These values suggest that the linear distances between all three landmarks increased with age, in both males and females. The annual changes in the linear distances between the bilateral skeletal landmarks are shown in Table 3. The overall average annual change was 0.5 mm for GPFd, 0.3 mm for NCd, and 0.7 mm for IOFd. Hence, the change was largest for IOFd and smallest for NCd. The *p*-values demonstrated that the annual change of each variable studied was statistically significant. Males had greater annual changes than females for GPFd and IOFd, but not for NCd.

**Table 2:** Descriptive statistics of continuous dependent variables.

Variable	Male (n = 50)		Female (n = 50)		Overall (n = 100)	
	Mean ± SD	Range	Mean ± SD	Range	Mean ± SD	Range
GPFd at T1 (mm)	31.0 ± 2.3	25.9–35.9	29.4 ± 2.5	24.5–35.8	30.2 ± 2.5	24.5–35.9
GPFd at T2 (mm)	32.3 ± 2.4	27.4–37.8	30.2 ± 2.5	24.7–36.6	31.2 ± 2.7	24.7–37.8
NCd at T1 (mm)	28.0 ± 3.6	21.2–35.6	26.4 ± 3.1	15.7–33.0	27.2 ± 3.4	15.7–35.6
NCd at T2 (mm)	28.9 ± 3.7	21.4–38.5	27.0 ± 2.9	16.4–32.8	27.9 ± 3.5	16.4–38.5
IOFd at T1 (mm)	52.6 ± 4.0	45.0–62.0	51.4 ± 3.8	42.8–61.6	52.0 ± 3.9	42.8–62.0
IOFd at T2 (mm)	54.7 ± 4.2	45.9–64.9	52.7 ± 3.7	44.9–61.8	53.7 ± 4.0	44.9–64.9

SD: Standard deviation.

GPFd: Transverse linear distance between greater palatine foramina.

NCd: Transverse linear distance between nasal cavity walls.

IOFd: Transverse linear distance between infraorbital foramina.

**Table 3:** Annual changes in linear distances between bilateral skeletal landmarks.

Variable	Male	Female	Overall
∆GPFd/year (mm)	0.6 ± 0.3**	0.3 ± 0.3**	0.5 ± 0.3**
∆NCd/year (mm)	0.3 ± 0.4**	0.3 ± 0.5*	0.3 ± 0.5**
∆IOFd/year (mm)	0.8 ± 0.5**	0.5 ± 0.3**	0.7 ± 0.4**

Results are mean Values ± standard deviations. *p < 0.001; **p < 0.0001, indicating statistically significant annual changes.

GPFd: Transverse linear distance between greater palatine foramina.

NCd: Transverse linear distance between nasal cavity walls.

IOFd: Transverse linear distance between infraorbital foramina.

**Figure 5:**
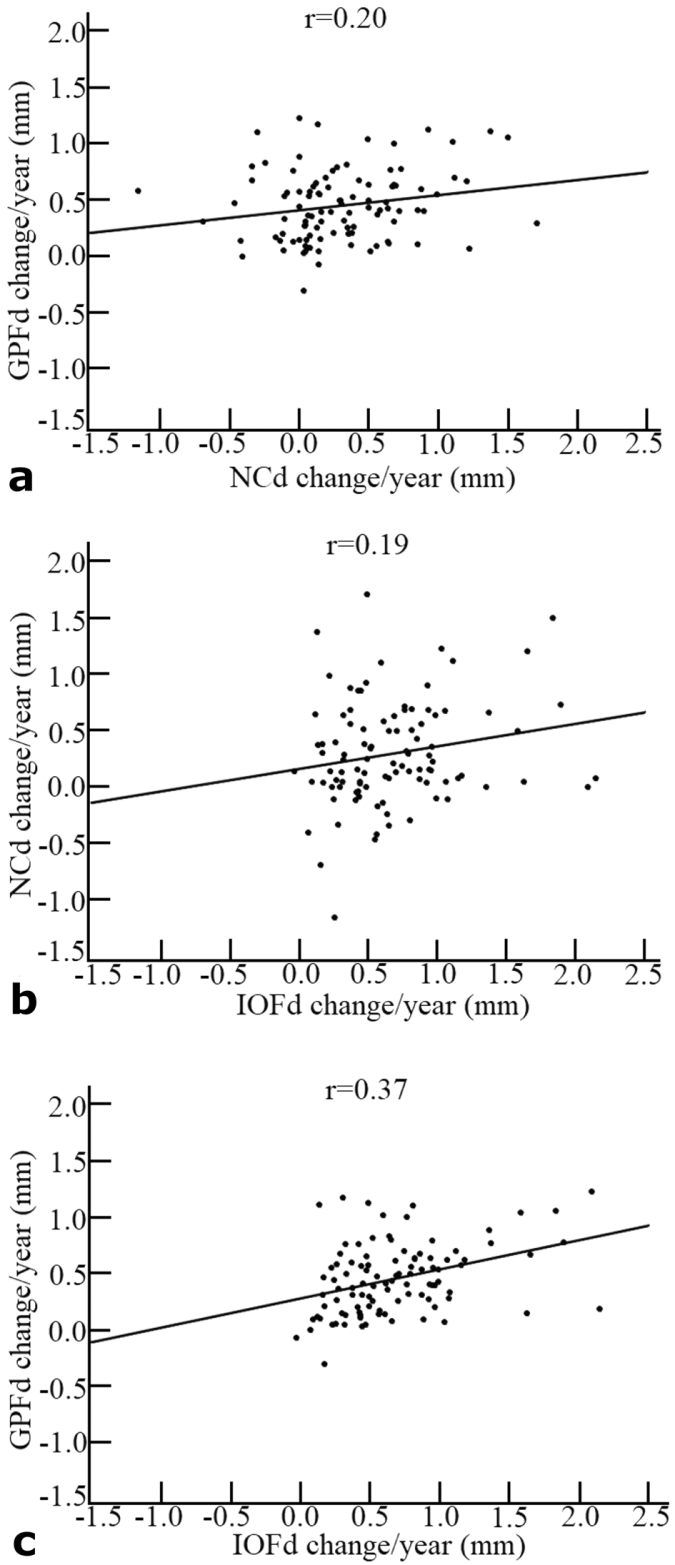
Scatter plot matrices demonstrating how each dependent variable changed in relation to the others. Correlation between annual changes of (**a**) distance between lateral margins of the greater palatine foramina (GPFd) and distance between lateral walls of the nasal cavity (NCd), (**b**)NCd and distance between lateral margins of the infraorbital foramina (IOFd), and (**c**) GPFd and IOFd.

Pearson’s correlation coefficients demonstrated weak, statistically not significant (*p* > 0.05) correlations between age at T1 and the annual change in the dependent variables (Table 4). The scatter plot matrices shown in Fig. 5 demonstrate how each dependent variable changed in relation to the other dependent variables. Although all linear correlations were relatively weak, both the correlation between annual GPFd change and annual NCd change (*p* = 0.047) and the correlation between annual GPFd change and annual IOFd change (*p* < 0.001) were statistically significant.

**Table 4:** Pearson’s correlation coefficients (r) describing the relationships between age at T1 and the annual change of each variable measured.

Variable	Male		Female		Overall	
	r	*P*	r	*p*	r	*p*
GPFd	−0.17	0.23	0.12	0.39	0.10	0.30
NCd	0.08	0.60	−0.04	0.78	0.05	0.65
IOFd	−0.01	0.92	−0.13	0.37	0.08	0.42

No statistically significant correlations (*p* > 0.05).

GPFd: Transverse linear distance between greater palatine foramina.

NCd: Transverse linear distance between nasal cavity walls.

IOFd: Transverse linear distance between infraorbital foramina.

**Figure 6:**
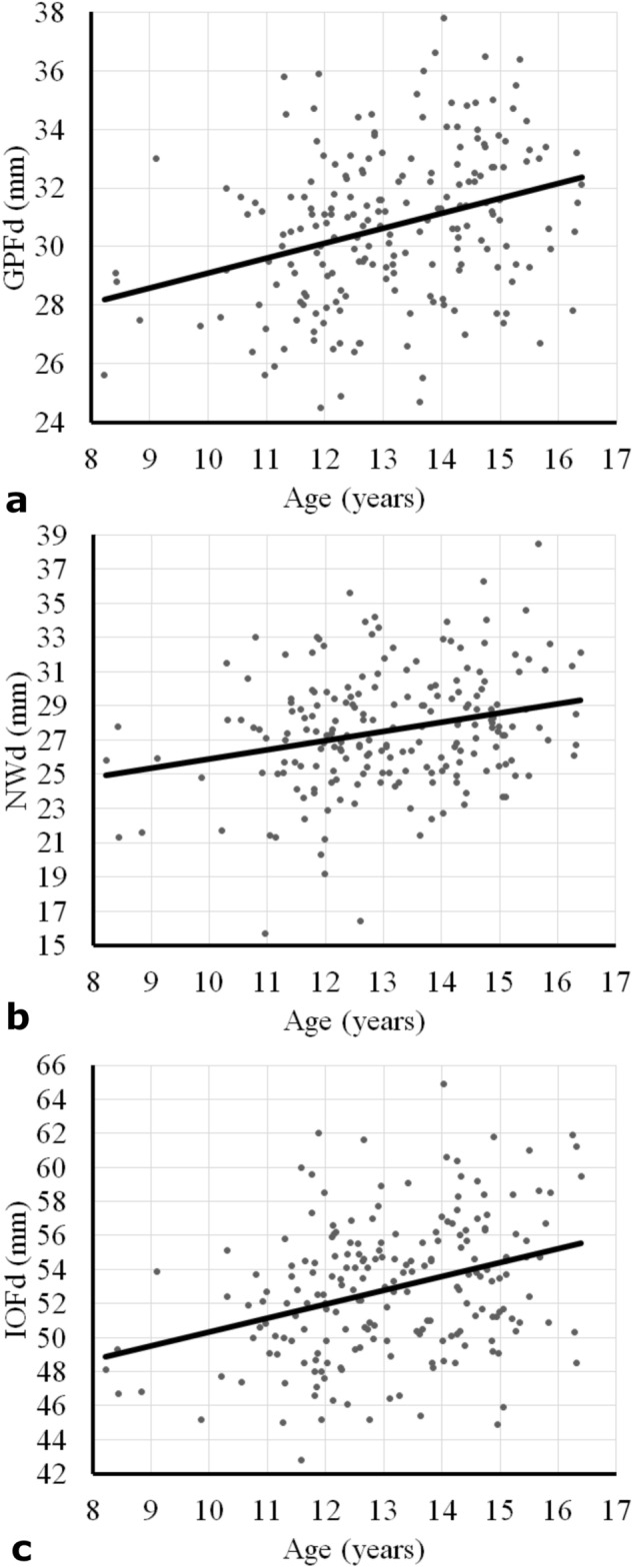
Scatter plots with regression lines illustrating the average dimension by age. (**a**) distance between lateral margins of the greater palatine foramina (GPFd), (**b**) distance between lateral walls of the nasal cavity (NCd), and (**c**) distance between lateral margins of the infraorbital foramina (IOFd).

The distances determined for GPFd, NCd, and IOFd plotted against the patient age at which a CBCT was taken are shown in Fig. 6 to illustrate the changes in the transverse dimension that occurred with age.

## Discussion

Our principal finding was that the transverse distance between all three maxillary landmarks studied increased with age. Hence, the null hypothesis that these landmarks do not change with growth is rejected. Although transverse widening of the maxilla during growth has been described previously, and the changes as such are not unexpected, the present study is the first to quantify these changes using 3D imaging data and three sets of easily identifiable skeletal landmarks at various vertical levels of the maxilla. While the average annual change was 0.5 mm for GPFd, 0.3 mm for NCd, and 0.7 mm for IOFd in the overall sample, the annual increases in GPFd and IOFd were significantly greater in males than in females. Although the peak height velocity of females lies within the age range of the patients studied, while that of males lies somewhat outside of that range^8,9^, this finding is barely surprising. Females are generally smaller in stature and it is logical that their average changes with growth would be proportionally smaller, too. The annual transverse change was not statistically associated with age at T1 for any of the variables measured. This means that there was no clear growth peak for the landmarks studied. This corroborates the findings of other studies, which suggest that chronologic age and other biological indicators do not always correlate with skeletal or facial growth status and that sometimes a real peak may be missing^8–13^. It is also of note that craniofacial structures grow proportionally less than structures further away from the head^14^. This may, at least in part, explain the small annual increases in the distance between bilateral structures in the facial area found in the present study.

A recent study on a novel predictor of skeletal response to maxillary expansion used the same landmarks as in our study to quantify long-term effects of RME treatment^15^. In contrast to previous reports of pyramidal expansion with RME^16^, the authors found a larger proportion of expansion at the level of the infraorbital foramina than at the more caudal levels of greater palatine foramina and nasal cavity^15^. Our study showed similar changes with natural growth—a larger increase at a more cranial level: the IOFd increased an average of 0.7 mm per year, whereas the GPFd and NCd increased 0.5 and 0.3 mm per year, respectively. This suggests that measurements using the infraorbital foramina as landmarks are significantly influenced by growth, which must be considered when the outcome of expansion treatment is assessed. Prior to this study, little was known about how the foramina of the maxilla change during the prepubertal period.

Some growth studies have demonstrated that the cortical bone lining the inner surface of the nasal cavity undergoes periosteal surface removal of bone as its endosteal side receives simultaneous deposits of new bone, contributing to an increase in nasal width^17^. The present study sheds more light on the rate of this increase during adolescence.

The greater palatine foramina are located in the hard palate. Our results suggest that they move laterally as the palatine process of the maxilla grows. The transverse growth occurs mainly by midpalatal suture separation, which is greater posteriorly than anteriorly^16,18^. The classic implant studies by Björk and Skieller suggest that this transverse growth occurs at 0.42 ± 0.12 mm per year^18,19^. Similar growth rates were reported by Korn and Baumrind with 0.51 ± 0.16 mm per year^20^. The present results correspond well with the classic implant studies and suggest an annual growth rate of 0.50 ± 0.31 mm for the distance between the greater palatine foramina (GPFd). It was hitherto not known if the results of the classic growth studies could be applied to foramina or other bony landmarks in the maxilla.

It would be logical to assume that the landmarks associated with the maxilla would change concurrently in the transverse dimension. However, the correlations among the annual changes in the distances between the landmarks were relatively low. These findings suggest that the growth of the maxilla cannot be simplified to assume that all landmarks grow simultaneously or by the same amount. This corroborates Enlow’s postulate that growth proceeds in a complex variety of directions in some major regions of the maxilla^17^.

Our findings have potentially valuable clinical applications. The most common transverse problem in orthodontics is a narrow maxilla, which may cause posterior crossbites. Identifying a crossbite as a transverse problem in patients is not difficult; however, there is little data available to help clinicians quantify the amount of skeletal transverse deficiency of a patient. The data obtained in this study, taken from a large sample of growing dental Class I subjects, may reflect population averages of transverse distances between landmarks. Clinicians may use this data as a diagnostic tool to help them quantify the transverse skeletal problems of their patients.

The treatment of choice for a maxillary transverse skeletal problem is often rapid maxillary expansion (RME) to open the midpalatal suture^21,22^. When a tooth-borne expander is used, the heavy forces generated by the expander transmit through the teeth into the maxillary bones and separate the hemimaxillae, which leads to subsequent bone deposition at the suture. This skeletal expansion occurs together with dentoalveolar expansion in the form of dental tipping and alveolar bending^22–27^. Quantifying the amount of skeletal expansion has previously been difficult because RME is typically performed on preadolescent patients so long-term effects are a combination of treatment and naturally occurring growth. Up to now, our understanding of maxillary skeletal growth was based mainly on decades-old studies using implants, frontal cephalograms, and dental model measurements with all their limitations^18–20,28^. Confirming that the transverse growth rate at the greater palatine foramina is similar to the maxillary growth reported in the classic implant studies is helpful in allowing clinicians to more confidently estimate the amount of long-term skeletal expansion achieved with RME.

Some limitations of the present study must be considered when interpreting its results. The study population was a convenience sample of patients who had undergone orthodontic treatment. As a result, the CBCT scans were acquired based on clinical need rather than research purposes. For this reason, not all patients were of the same age at the time of the initial CBCT scan or had a standardized time period between T1 and T2. Consequently, we reported any change with growth as average change per year, not as a total amount. In addition, although unlikely in a Class I dento-skeletal sample, the presence of fixed orthodontic appliances might have influenced the development of the maxillary transverse dimension. The ideal study sample would have been an untreated Class I sample with serial CBCT scans and no history of orthodontic treatment. However, such a sample is impossible to find, as it would be unethical to expose individuals to ionizing radiation without diagnostic or therapeutic benefit. After all, the guiding principle of radiation safety is “ALARA,” which stands for “as low as reasonably achievable.” This principle means that even if it is a small dose, if receiving that dose has no direct benefit, you should try to avoid it.

On the other hand, the present study has some substantial strengths. Measurements were performed on a total of 100 subjects, a sample size substantially larger than in most other studies that evaluated the transverse dimension of the maxilla, making its results generalizable to a larger population. In addition, CBCT allowed visualization of the whole maxillofacial complex without any magnification or superimposition of anatomic structures commonly associated with two-dimensional images^2,3^.

In conclusion, the present results show that the distances between the greater palatine foramina, lateral walls of the nasal cavity, and infraorbital foramina increase significantly during growth suggesting that the positions of these landmarks change in conjunction with the transverse growth of the maxilla. The annual change is generally smallest for the NCd and largest for IOFd with males having greater annual changes than females for GPFd and IOFd, but not NCd. The growth rates found in this study provide normative data on maxillary transverse growth, which can serve as population average and may allow orthodontic clinicians to more confidently estimate the amount of skeletal transverse deficiency or evaluate the long-term effects of skeletal expansion treatment.

## References

[1] Moyers RE, Bookstein FL (1979). The inappropriateness of conventional cephalometrics. Am. J. Orthod..

[2] De Vos W, Casselman J, Swennen GRJ (2009). Cone-beam computerized tomography (CBCT) imaging of the oral and maxillofacial region: A systematic review of the literature. Int. J. Oral Maxillofac. Surg..

[3] Noar J, Pabari S (2013). Cone beam computed tomography–current understanding and evidence for its orthodontic applications?. J. Orthod..

[4] Schlicher W, Nielsen I, Huang J, Maki K, Hatcher D, Miller AJ (2012). Consistency and precision of landmark identification in three-dimensional cone beam computed tomography scans. Eur. J. Orthod..

[5] Berco M (2009). Accuracy and reliability of linear cephalometric measurements from cone-beam computed tomography scans of a dry human skull. Am. J. Orthod. Dentofac. Orthop..

[6] Lagravère MO, Carey J, Toogood RW, Major PW (2008). Three-dimensional accuracy of measurements made with software on cone-beam computed tomography images. Am. J. Orthod. Dentofac. Orthop..

[7] Koo TK, Li MY (2016). A guideline of selecting and reporting intraclass correlation coefficients for reliability research. J. Chiropr. Med..

[8] Hägg U, Taranger J (1980). Skeletal stages of the hand and wrist as indicators of the pubertal growth spurt. Acta Odontol. Scand..

[9] Mellion ZJ, Behrents RG, Johnston LE (2013). The pattern of facial skeletal growth and its relationship to various common indexes of maturation. Am. J. Orthod. Dentofacial Orthop..

[10] Baccetti T, Franchi L, McNamara JA (2005). The Cervical Vertebral Maturation (CVM) method for the assessment of optimal treatment timing in dentofacial orthopedics. Semin. Orthod..

[11] Fishman LS (1982). Radiographic evaluation of skeletal maturationA clinically oriented method based on hand-wrist films. Angle Orthod..

[12] Ochoa BK, Nanda RS (2004). Comparison of maxillary and mandibular growth. Am. J. Orthod. Dentofacial Orthop..

[13] Fishman LS (1979). Chronological versus skeletal age, an evaluation of craniofacial growth. Angle Orthod..

[14] Proffit WR (2013). Contemporary Orthodontics.

[15] Grünheid T, Larson CE, Larson BE (2017). Midpalatal suture density ratio: A novel predictor of skeletal response to rapid maxillary expansion. Am. J. Orthod. Dentofacial Orthop..

[16] Bazargani F, Feldmann I, Bondemark L (2013). Three-dimensional analysis of effects of rapid maxillary expansion on facial sutures and bones. Angle Orthod..

[17] Enlow DH, Bang S (1965). Growth and remodeling of the human maxilla. Am. J. Orthod..

[18] Björk A, Skieller V (1974). Growth in width of the maxilla studied by the implant method. Scand. J. Plast. Reconstr. Surg..

[19] Björk A, Skieller V (1977). Growth of the maxilla in three dimensions as revealed radiographically by the implant method. Br. J. Orthod..

[20] Korn E, Baumrind S (1990). Transverse development of the human jaws between the ages of 8.5 and 15.5 years, studied longitudinally with use of implants. J. Dent. Res..

[21] Haas A (1961). Rapid expansion of the maxillary dental arch and nasal cavity by opening the mid-palatal suture. Angle Orthod..

[22] Lagravère MO, Major PW, Flores-Mir C (2005). Long-term skeletal changes with rapid maxillary expansion: A systematic review. Angle Orthod..

[23] Bressane LB, Janson G, Oltramari-Navarro PVP, Henriques JFC, Garib DG (2016). Long-term changes of alveolar buccal bone after rapid maxillary expansion in an adolescent patient. J. World Fed. Orthod..

[24] Ghoneima A (2011). Effects of rapid maxillary expansion on the cranial and circummaxillary sutures. Am. J. Orthod. Dentofacial Orthop..

[25] Bishara SE, Staley RN (1987). Maxillary expansion: clinical implications. Am. J. Orthod. Dentofacial Orthop..

[26] Martina R (2012). Transverse changes determined by rapid and slow maxillary expansion—A low dose CT based randomized controlled trial. Orthod. Craniofac. Res..

[27] McNamara JA (2006). Long-term adaptations to changes in the transverse dimension in children and adolescents: An overview. Am. J. Orthod. Dentofacial Orthop..

[28] Hesby RM (2006). Transverse skeletal and dentoalveolar changes during growth. Am. J. Orthod. Dentofacial Orthop..

